# Artificial Intelligence Segmented Dynamic Video Images for Continuity Analysis in the Detection of Severe Cardiovascular Disease

**DOI:** 10.3389/fnins.2020.618481

**Published:** 2021-02-10

**Authors:** Xi Zhu, Wei Xia, Zhuqing Bao, Yaohui Zhong, Yu Fang, Fei Yang, Xiaohua Gu, Jing Ye, Wennuo Huang

**Affiliations:** ^1^Clinical Medical College, Yangzhou University, Yangzhou, China; ^2^Department of Computer Science and Technology, Nanjing University, Nanjing, China

**Keywords:** artificial intelligence, segmented dynamic, video imaging, detection of severe cardiovascular disease, continuity analysis

## Abstract

In this paper, an artificial intelligence segmented dynamic video image based on the process of intensive cardiovascular and cerebrovascular disease monitoring is deeply investigated, and a sparse automatic coding deep neural network with a four layers stack structure is designed to automatically extract the deep features of the segmented dynamic video image shot, and six categories of normal, atrial premature, ventricular premature, right bundle branch block, left bundle branch block, and pacing are achieved through hierarchical training and optimization. Accurate recognition of heartbeats with an average accuracy of 99.5%. It provides technical assistance for the intelligent prediction of high-risk cardiovascular diseases like ventricular fibrillation. An intelligent prediction algorithm for sudden cardiac death based on the echolocation network was proposed. By designing an echolocation network with a multilayer serial structure, an intelligent distinction between sudden cardiac death signal and non-sudden death signal was realized, and the signal was predicted 5 min before sudden death occurred, with an average prediction accuracy of 94.32%. Using the self-learning capability of stack sparse auto-coding network, a large amount of label-free data is designed to train the stack sparse auto-coding deep neural network to automatically extract deep representations of plaque features. A small amount of labeled data then introduced to micro-train the entire network. Through the automatic analysis of the fiber cap thickness in the plaques, the automatic identification of thin fiber cap-like vulnerable plaques was achieved, and the average overlap of vulnerable regions reached 87%. The overall time for the automatic plaque and vulnerable plaque recognition algorithm was 0.54 s. It provides theoretical support for accurate diagnosis and endogenous analysis of high-risk cardiovascular diseases.

## Introduction

Chronic non-communicable diseases (NCDs) have become a major public health problem affecting the country’s economic and social development, with mortality accounting for 86.6% of the entire spectrum of diseases, and the burden of the disease accounting for more than 70% (Luo et al., 2018). Among the hazards of chronic diseases, cerebrovascular, and cardiovascular diseases (CCVD) have been occupying the first place in the whole spectrum of diseases in terms of morbidity and mortality, and the prevalence rate is on the rise (Bechar et al., 2018). Electrocardiogram (ECG) and medical imaging are the main tools for clinical diagnosis of cardiovascular disease. ECG includes a short-time electrocardiogram (EKG) collected at rest and a long-time dynamic electrocardiogram (Holter) collected under moving conditions (Sonkusare et al., 2019). The main advantages of ECG are that it is easy, inexpensive, and portable to collect electrical signals from outside the body and that it responds to the electrophysiological activity of the heart (Nascimento and Carneiro, 2017). Compared with electrocardiogram, it can reveal the internal activity of the heart. Its disadvantages are the fixed collection position, long collection time, high price, immobility, and the inability to realize real-time collection (Latha et al., 2020). Cardiovascular disease has both covert and sudden features. The covert nature of the disease determines that ECG cannot be observed only, and more accurate medical images are needed to discover hidden diseases and symptoms, to carry out timely intervention and treatment; the sudden nature of the disease also determines that medical images cannot be used only, and ECG needs to be portable to monitor the condition of the heart in real-time and provide timely warning of possible dangers (Randive et al., 2020). Only when ECG and medical imaging cooperate, can the mortality rate of cardiovascular disease be effectively reduced (Musial et al., 2020). However, the existing dynamic ECG equipment still adopts offline storage mode, which cannot realize timely signal analysis and early warning; medical image analysis is limited by the professional level of doctors themselves, and at the same time is easily interfered by other factors, which can easily produce inconsistent results among different doctors, and cannot achieve the purpose of accurate diagnosis and treatment (Aminikhanghahi and Cook, 2017). Therefore, the research on automatic analysis algorithms of ECG and medical images based on artificial intelligence plays a crucial role in the prevention and treatment of cardiovascular diseases (Darwish et al., 2020).

In arrhythmia analysis, Costa et al. (2019) proposed to extract arrhythmia-related features from the double-tree complex wavelet transform coefficients and classify them by multilayer neural network, which has higher sensitivity than discrete wavelet transform coefficients. Shinbane and Saxon (2018) proposed to extract RR interval features, higher-order statistical features, and Gaussian mixed model parameters from ECG signals, which is more sensitive than discrete wavelet transform coefficients. Characteristics to classify arrhythmias, and by training the decision tree model, arrhythmias were identified with an accuracy of 99.7%. In the diagnosis of premature ventricular beats, Xiao et al. (2019) proposed a fractional linear prediction method for the detection of premature ventricular beats and demonstrated that it has higher accuracy than linear prediction and a higher sensitivity to premature ventricular beats than other beats. Ventricular premature heartbeat, establish monitoring statistical model by discrete wavelet decomposition, and set the upper limit value of the model, when the monitoring statistical model parameters exceed the upper limit value, to alarm (Haider et al., 2019). When the parameters of the monitoring statistical model exceed the upper limit, the alarm will be raised (Fan et al., 2019). The accuracy of premature ventricular beats detection is 97.9% as verified by MIT-BIH arrhythmia database data (Deldari et al., 2020). In the aspect of myocardial infarction prediction, Yang et al. (2020) proposed a convolutional neural network method to identify myocardial infarction heartbeat and normal heartbeat in the ECG signal, and the recognition accuracy reached 93.53 and 95.22% through the noise-free environment and noise-free environment tests, respectively. Saggi and Jain (2018) also applied the convolutional neural network to automatically identify calcification in the coronary artery in coronary CT images. With a plaque recognition accuracy of 85% for images with cardiac motion disturbances (Saggi and Jain, 2018). However, due to the limitations of CT image acquisition and its resolution, it is not able to accurately analyze the intracoronary plaque composition, hence the emergence of endovascular medical images such as VIES and OCT (Hong et al., 2018). In contrast, Craye et al. (2016) extracted 54 sets of features from VIES images to describe fibrotic lipids, calcifications, necrotic cores, and fibrotic plaques in blood vessels and performed feature screening by PCA algorithm to achieve accurate classification of intravascular plaques. Different migratory learning methods used to establish different forms of image expressions, and eventually, the accurate detection of plaque tissue was achieved by fusing multiple representations, with a model detection accuracy of 91.7% (Craye et al., 2016). Pasterkamp also extracted a series of plaque-related geometric and non-geometric features by taking advantage of the high-resolution characteristics of coronary OCT images, and then applied the SVM classifier to classify the relevant features to achieve accurate identification of fibrotic, lipid, and calcified plaques, with an average identification accuracy of 94.0, 97.2, and 99.2%, respectively (Pasterkamp, 2018).

The main task of video image behavior recognition is to analyze a video image and then classify the human behaviors contained in it. The difficulty in video image behavior recognition comes from two aspects: first, the time variability, there may be gaps between the actions that have nothing to do with the behavior, the time point of the behavioral actions is uncertain, and the continuous interval between the actions is also different. Therefore, given a video image to be able to identify the start and end time of the behavior, for the video image frames that have nothing to do with the behavior, weaken its role or discard. Second, the spatial complexity, different perspectives, illumination, and background will cause different scenes, different scenes of the same behavior action will produce certain differences. Even in fixed scenes, behavioral actions can vary depending on the perspective of the person, individual differences, and shading. These can have an impact on accuracy. Current algorithms are dedicated to solving the problem of how to extract better features that describe the judgments made in video images and better temporal information in video images. Mainstream methods are using partial continuous frames of video images and long duration video image information, which results in a lot of missing information and there is a lot of redundant information in video images, and randomly selecting video image frames may miss a lot of important information. So, in this paper, the attention mechanism is used to empower the video image frames to weaken the redundant information and increase the useful video image frames to influence the result. In medical imaging, the coronary vascular morphology assessment and plaque identification algorithms closely related to high-risk cardiovascular diseases were investigated. The automatic endovascular contour extraction algorithm in coronary OCT images was investigated to achieve accurate extraction and 3D modeling of coronary vessels, and the adaptiveness of the coronary endovascular extraction algorithm to different feature images was realized through the grayscale distribution analysis of OCT images, which supported the accurate assessment of coronary vessel morphology.

## Artificial Intelligence Segmented Dynamic Video Images in Continuity Analysis Design

### Artificial Intelligence Modeling

In terms of the composition of the entire autoencoder network, the implicit layer can be viewed as a representation of features extracted from the input layer, and then the input is reproduced from these features by way of reverse coding (Banerjee et al., 2019). Therefore, the dimensionality of the implicit layer is usually smaller than the dimensionality of the input layer. The process of training the network is the process of continually reducing the error between the input and output, so the loss function of the network can be defined as:

(1) $$K\,\left(W,b\right)=\frac{1}{2\,p}\,\sum_{i=1}^{p}{||X^{i}-Y^{i}||}_{2}^{2}+\frac{\lambda }{2}\,\left({||W_{e}||}_{2}^{2}+{||W_{d}||}_{2}^{2}\right)$$

The first term is the mean-variance of the input and output data, and the second term is the attenuation term of the encoding and decoding network weight parameters, which is mainly used to reduce the update rate of the weights and prevent the data training from converging to the local optimum, n is the number of input samples, and mm is the attenuation coefficient of the weights (Diehn et al., 2016).

**FIGURE 1:**
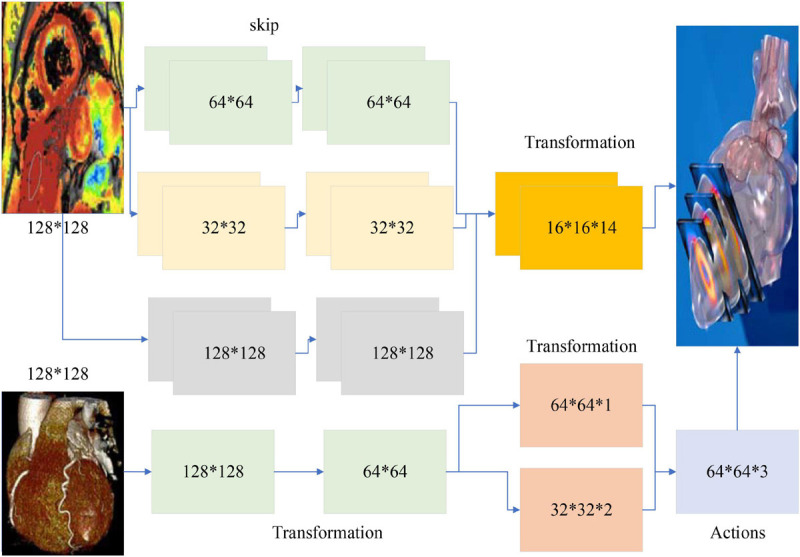
Artificial intelligence model architecture.

Different tasks have different feature complexity, so different numbers of implicit layer nodes are often required when applying autoencoder networks to solve practical problems (Eldib et al., 2018). The characteristic of the autoencoder implicit layer dimension is lower than the input data dimension can sometimes limit its application. To solve this problem, sparse rules are added to the autoencoder network. Sparsity is the selective activation of a small number of nodes in the implicit layer nodes, leaving most nodes in a suppressed state (Ali et al., 2018). This allows the number of implicit layer nodes in the network to be greater than the number of input layer nodes, and depending on the complexity of the task, different levels of sparsity can be set to meet the practical requirements, as shown in Figure 1.

Thus, an average activation function is introduced into the auto-coding network (Salekin et al., 2018), defined as:

(2) $$\bar{p_{j}}=\frac{1}{p}\,\sum_{i=1}^{p}H_{i}\,\left(X^{j}\right)$$

To ensure that the activation of the implicit layer nodes is small, a relative entropy function is introduced into the model, which is expressed as follows:

(3) $$PL\left(p||\bar{p_{j}}.\right)=p\log\frac{p}{\bar{p_{j}}}+\left(1-p\right)\log\frac{1-p}{1-\bar{p_{j}}}$$

The network training process is the process of progressively updating the network weight parameters, propagating to the implicit layer to get tr, X through the formula (3) propagates back to the data layer to get w, w again passed to the implicit layer to get T, then the formula for updating the network weight parameters can be expressed as.

(4) $$t\,r\,\left(w^{T}\,X\,L_{N}\,{\text{X}}^{\text{Tw}}\right)=t\,r\,\left(w^{T}\,X\,L\,{\text{X}}^{\text{Tw}}\right)$$

After completing the training of one layer of autoencoder, the feature representation in the implicit layer is obtained, and the feature representation obtained in this layer is used as the data input for the next layer, and the next layer of the network is trained to obtain the features of the next implicit layer, and so on to complete the training of the entire stack sparse autoencoder network layer by layer (Horton et al., 2020).

In this paper, a stack sparse autoencoder network with four layers is constructed, and the corresponding nodes from the first to the fourth layer are 120, 60, 30, and 15, respectively. The sparse autoencoder of the first layer is trained by the input heartbeat, and the feature output of the first layer is obtained. Then, the sparse autoencoder of the second layer is trained with the output of the first layer to obtain the second layer’s features. Finally, the four layers of sparse autoencoders are trained, and the depth feature of the cardioid signal is obtained. The depth features of the cardioid signal are extracted automatically during the network training, which solves the problem of incomplete features or redundancy between features when they are selected manually.

The error proportion from the feature implicit layer to the data output layer first calculated, which is expressed as:

(5) $$\sigma_{i}^{2}=(\left(\sum_{j=1}^{S}W_{i\,j}^{\left(2\right)}\sigma_{j}^{\left(3\right)}\right)+\beta \left(-p/\bar{p_{j}}+\left(1-p\right)/\left(1-\bar{p_{j}}\right)\right)$$

A direct transfer function corresponds to the physiological system of viewing aortic blood pressure as input and radial blood pressure as output, and to be able to reconstruct central arterial pressure from the radial arterial sphygmomanometer signal, an inverse transfer function would be derived from the above equation.

(6) $$P\,\left(t-1\right)=\frac{-b_{2}\,P\,\left(t-2\right)}{b_{1}}-\ldots \,\frac{-b_{n\,b}\,P\,\left(t-n\,b\right)}{b_{1}}$$

Fisher vector is an encoding method that enables the normalization of unequal feature matrices. Existing classification methods fall into two main categories: generative methods, such as GMM (Lee et al., 2019), which reflect the similarity between similar data, and discriminative methods, such as SVM, which reflect the differences between dissimilar data. The two advantages that work better will be used to generate models for use in the discriminant classifier.

(7) $$\gamma_{t}\,\left(i\right)=p\,\left(i|\lambda_{t},\lambda \right)=\frac{w_{i}.p_{i}\,\left(\lambda_{t}|\lambda \right)}{\sum_{i=1}^{M}w_{i}.p_{i}\,\left(\lambda_{t}|\lambda \right)}$$

A first bias derivation of the parameters yields (Ren et al., 2019):

(8) $$\frac{\partial L\,\left(\lambda_{t}|\lambda \right)}{\partial w_{i}}=\sum_{i=1}^{T}\left[\frac{\gamma_{i}\,\left(i\right)}{w_{i}}-\frac{\gamma_{t}\,\left(1\right)}{w_{i}}\right],i>1$$

(9) $$\frac{\partial L\,\left(\lambda_{t}|\lambda \right)}{\partial w_{i}^{d}}=\sum_{i=1}^{T}\gamma_{t}^{i}\,\left[\frac{\gamma_{i}\,{\left(i\right)}^{2}}{w_{i}}-\frac{\gamma_{t}\,{\left(1\right)}^{2}}{w_{i}}\right]$$

(10) $$\frac{\partial L\,\left(\lambda_{t}|\lambda \right)}{\partial w_{i}^{d}}=\sum_{i=1}^{T}\gamma_{t}^{i}\,\left[\frac{\gamma_{i}\,{\left(i\right)}^{2}}{w_{i}}-\frac{1}{w_{i}}\right]$$

The optical flow field is used to obtain the trajectory information in the video sequence: to sample the feature points densely at multiple scales of the picture, respectively, a partition grid is used to filter out the points with few transformations; to obtain the motion velocity of the feature points, the median optical flow in the neighborhood of the feature points is computed, and then the key points are tracked; a total of four types of HOG, HOF, trajectory, and MBH are extracted along with the trajectory information. The HOG feature is based on the grayscale image calculation, and several other features are based on the dense optical flow field calculation. The HOG feature is the gradient amplitude of a pixel counted according to its gradient direction after blocking. The HOF feature is obtained by calculating the grayscale transformation matrix and gradient matrix of the optical flow adjacent to the current frame at a time, and then weighting the optical flow direction.

The most important improvement is the use of camera motion estimation to remove trajectories and optical flows present in the background. In the DT algorithm, as the camera moves, it generates many trajectories on the background, and this motion information can greatly affect the trajectory of the human body. This trajectory information is noisy and has a weak relationship with the identified behavior. So, there is a need to eliminate this type of noise. The motion of the trajectory is calculated by computing the optical flow information, so to eliminate the background the optical flow can be estimated by estimating the camera movement. Since the variation between two adjacent frames is small, the algorithm assumes that a projection transformation matrix is used to describe the relationship between two adjacent images, i.e., the latter frame is transformed by the projection of the previous frame. Thus, the estimation of camera motion becomes a matter of computing the transformation projection matrix from the images of the previous and previous frames.

### Segmented Moving Video Images in Continuity Analysis Design

**FIGURE 2:**
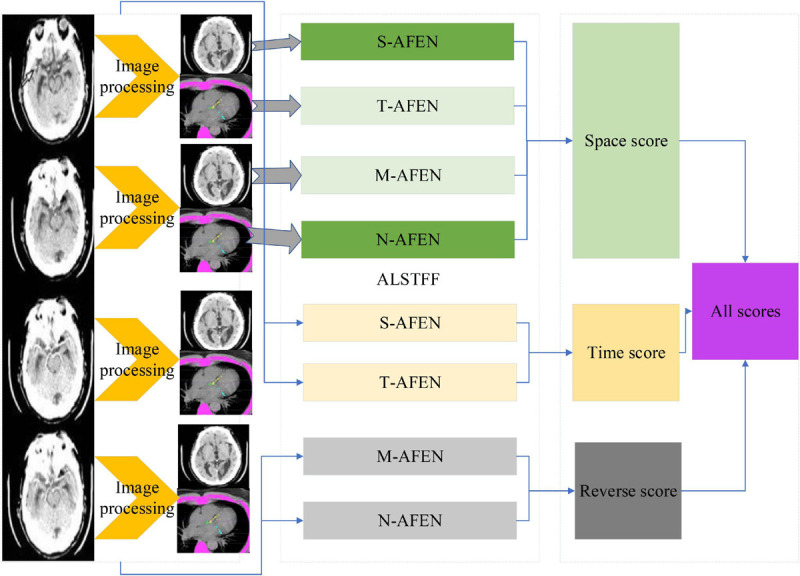
ALSTFF overall flow chart.

The classical dual-stream network model is divided into a temporal network, where the input to the temporal network is a stack of optical frames, and a spatial network, where the input to the spatial network is a single RGB image (Humphreys and Wang, 2018). This approach can only use a limited number of video frames in the video, but not the entire video information, besides, the spatial network input is a single random RGB image, which can only capture the appearance of a frame, there are some frames in the video that has nothing to do with the action, so it is a big mistake to extract appearance information from a random frame. The time correlation between successive frames is extracted by superimposed optical flow frames. For the convolutional network, if the number of convolutional kernels is much smaller than the number of superimposed optical flow frames, the information between frames is lost. The overall block diagram of the network is shown in Figure 2.

The input of the whole network is a long duration image sequence containing a motion, which is divided into several overlapping segments by video pre-processing. For each overlapping segment, it includes the RGB frame segment and optical stream frame segment, respectively, and the RGB frame segment is used as input to the spatial network; while the optical stream frame is a single channel, to keep the three-channel input of each sub-network consistent, the optical stream u, optical stream v and the mean value between them are used to form a three-channel picture, and the continuous optical stream frame is used as input to the temporal network (Mariakakis et al., 2017). The whole network is divided into several branches, and the layer number and hyper-parameter settings of each sub-network are the same before feature fusion, which can facilitate the training of the network, and also ensure that the features are consistent in dimension when fusing different stream features, which provides convenience for the fusion of different network features. The design idea of the network structure proposed in this paper is divided into five modules, the first module is the video pre-processing module, the second module is the convolutional neural network plus LSTM extraction of spatiotemporal features module, the third module is the attention mechanism module, the fourth module is the long duration information fusion module, and the fifth module is the classification module. The second and third module is called attentional mechanism feature extraction network, video frame by frame through the convolutional network to extract spatial features, through the LSTM decoding, adding attention mechanism in the decoding process to learn the weight of the impact of each frame on the results, and finally through the SoftMax layer output prediction results.

Video pre-processing module, the main function of this module is to process the original video and divide the input video into several clips. One-third of the length of each clip overlaps each other to maintain the continuity of the information. The input of the network is divided into RGB raw frame and optical stream frame, and the video is transformed into the corresponding picture to be saved by the coding and decoding method and optical stream algorithm in OpenCV.

Video pre-processing consists of two parts: segmentation and optical stream extraction. To extract the temporal and spatial features of the long-term video, we divide the input video into Sn fragments. One-third of the length of each fragment overlaps each other to maintain the continuity of the information. The input of the whole network is divided into two major pieces, RGB video frames, and optical stream frames (Jeannot, 2019). RGB frames can be read directly from the video in OpenCV and each frame of the video is saved as a picture. The optical stream frame is calculated by an algorithm that extracts the optical stream. In this paper, the TV-L1 algorithm is selected as the optical stream extraction algorithm.

**FIGURE 3:**
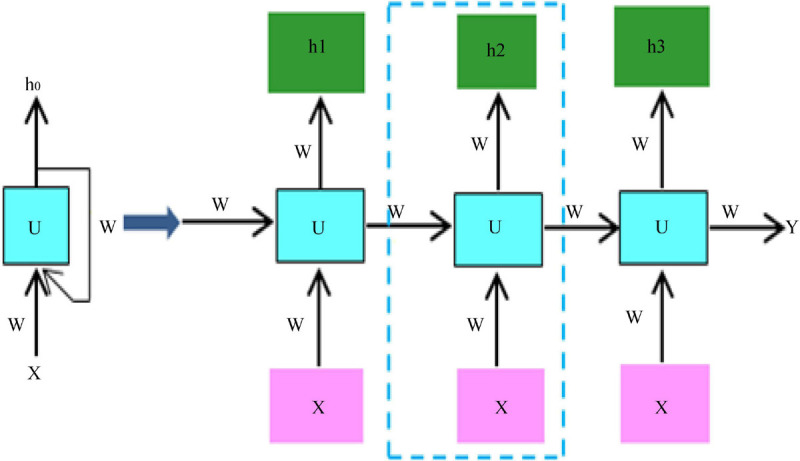
RNN structure diagram.

Due to the relatively small amount of data in the two datasets, using a small sample of data to train the network can easily lead to overfitting of the network. The current state-of-the-art neural networks require 1,000 of images to train the network to perform well (Eriksson and Eriksson, 2019). If there is not a lot of data, data augmentation is one of the ways to increase the amount of data, we do not have to look for novel images to add to the data set, because in neural network training, the recognition of images is not so intelligent, and inadequately trained neural network will think that the same object at different positions and angles belong to different objects, while in the eyes of humans, the object is only by displacement and change the angle. So, to get more data, just make small changes to the existing dataset, like rotation, displacement, flipping, etc. Our network will treat these images as different. Video belongs to a sequence problem which includes both spatial and temporal sequences, to solve such a problem we need to use RNN, the structure of RNN is shown schematically in Figure 3.

Video behavior recognition currently exists in algorithms, both manual feature-based, and deep learning-based methods, to extract spatial and temporal information from the video, spatial information contains information about the appearance of the video, and temporal information contains information about the temporal continuity between frames of the video. The spatial information is usually extracted using a convolutional neural network, and the temporal information is usually extracted using a cyclic neural network, which uses different filters to get different picture features. Different convolutional kernels correspond to different picture effects. The convolutional neural network can learn the features of the image in a supervised way, that is, it can learn the values inside the convolutional kernel, and through the backpropagation mechanism, the error is transmitted back, and the values in the convolutional kernel are updated at each layer by automatic derivation, which is the so-called gradient, and finally the error is minimized. At the same time, after each layer of convolution, all need to go through a non-linear activation function, adding non-linear factors in the neural network, so that the network can learn more complex functional characteristics, if you do not use non-linear activation function, then the output of the model is just a linear combination of the input, even if there are multiple hidden layers, if you use a linear activation function or no activation function, then the network has always done is linear. So, it is equivalent to having no hidden layer, the neural network just linearly combines the inputs and then outputs. The pooling operation is performed after the convolutional layer, which on the one hand reduces the dimensionality of the data, and at the same time reduces the redundant features in the image. Thus, theoretically the deeper the network, the richer the features that can be learned and the better the results.

## Analysis of the Process of Video Image Surveillance Detection of Severe Cardiovascular Disease

### Design of Video Imaging Tests for Critical Cardiovascular Disease

**FIGURE 4:**
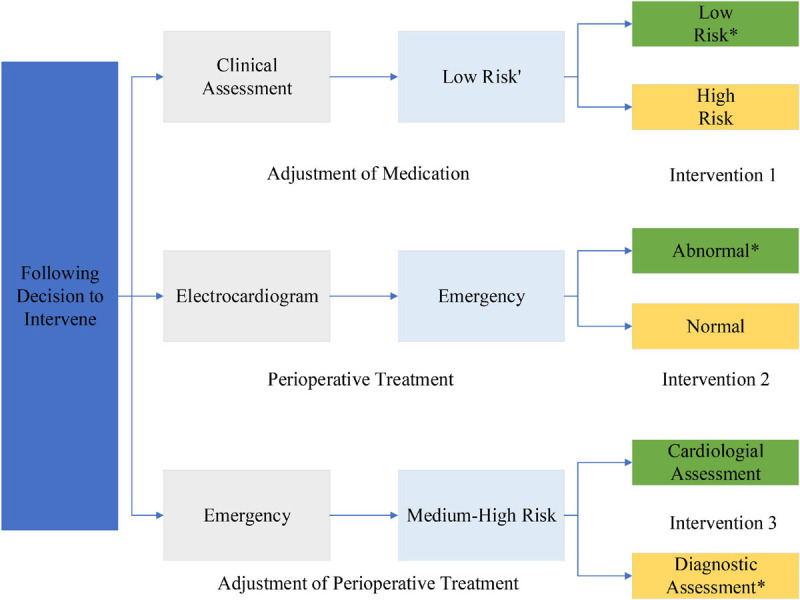
Video imaging test steps for severe cardiovascular and cerebrovascular disease.

Central arterial pressure measured by an invasive/minimally invasive device is used as the gold standard reference, and peripheral blood pressure measured by a non-invasive finger sphygmomanometer is reconstructed by an algorithm to evaluate the accuracy of the algorithm by comparing the central arterial pressure with its analysis error. The number of stable patients requiring interventional intubation (the head of the interventional guidewire is placed at 8 cm from the heart valve, or close to it) is more than or equal to 10. Experimental preparation phase: the operator prepares the experiment, including putting on clothing, gloves, and mask, placing the device in the appropriate position, and connecting the interface cable; the subject lies still on the experimental bed for 5 min; the subject wears the sensor component on the left finger; the continuous blood pressure measurement device is started until the finger blood pressure data can be read correctly, and The interventionalist inserts a micro-manometer catheter equipped with a special blood pressure measuring device into the aortic root by puncturing the femoral/radial/brachial artery and places the head of the catheter about 8 cm away from the heart valve, and the external sensor is collected by a data acquisition card and transferred to the computer for adjusting the blood pressure waveform at the central artery. After the waveform readings are stable and the absolute values are accurate, record the waveform data for 1 min; withdraw the probe and remove the peripheral blood pressure measurement equipment to complete the data acquisition for one patient, and repeat the above experimental steps for the other patients, as shown in Figure 4.

A total of seven minimally invasive surgery patients were collected, and their basic conditions are shown in Table 1. The time delay PTT of the blood pressure wave from the finger to the heart was obtained using the estimation method. This study was approved by the ethics committee and the patients provided consent forms.

**TABLE 1:** Statistics of experimental results.

**Patient number**	**RMSE (mmHg)**	**PTT (ms)**
1	3.4	34
2	4.1	24
3	4.7	75
4	5.7	77
5	5.9	83
6	5.5	42
7	3.5	72
Average value	2.9	71
Variance	5.8	60

The procedure consisted of each subject has his or her head slightly elevated and breathing steadily in a supine position for approximately 5 min before the start of the experiment. Continuous finger blood pressure was then monitored by a non-invasive blood pressure device at the same level as the heart for 5 min or more to ensure that the smooth muscle of the fingers adapted to the pressure of the finger cuff. A small homemade cuff is wrapped around the middle phalanx of the finger, as shown in Figure 4. Continuous finger blood pressure is recorded for 15 cardiac cycles before the inflation of the cuff. The cuff is then rapidly inflated to 200 mmHg to close the finger vessels, and finger blood pressure signals are recorded for an additional 15 cardiac cycles as the pressure wave reaches a steady state after inflation. The ipsilateral brachial blood pressure then measured using a sociometric blood pressure device as a reference to upper arm blood pressure. An analogy band-pass filter with cut off frequencies of 1 and 35 Hz was also used to obtain the ECG signal. The ECG signal also obtained from the ECG module. Finally, a data acquisition system was used to simultaneously acquire the finger blood pressure and ECG signals at a sampling rate of 1,000 Hz.

The acquired data were processed offline, and if the mean arterial pressure difference (MAP) between the reference BAP and the continuous FAP was less than 4 mmHg, the BAP was reconstructed using Eqs (8) or (9) and the recorded data, where parameter A in Eq. (10) takes the mean value from the literature. The data processing is divided into three parts: pre-processing, calculation of PTT, and reconstruction of upper arm blood pressure, as shown in Figure 4. In the pre-processing section, the collected waveform is processed by 20 Hz, 5-step Barth low-pass filtering, and then the time markers are used to identify the valid data segments, i.e., 15 cycles before and after artery closure. In the PTT calculation section, the apex of the ECG R-wave and the start of the finger blood pressure wave before arterial closure are identified, then the Pulse Arrival Time (PAT) value of a single cycle is calculated and averaged over 15 cycles, then the Pre-Ejection Period (PEP) value is subtracted to obtain the total PTT from the finger to the heart. Finally, the PTT value from the finger to the upper arm is calculated proportionally. To reconstruct the upper arm blood pressure, we first adopt the method that the blood pressure wave measured at the time of artery closure is equal to two times of the advance wave to get the advance wave of blood pressure; then we subtract the advance wave from the finger blood pressure wave measured before artery closure to get the reflection wave and reflection coefficient of blood pressure; then we find the true reflection coefficient according to the relationship between the reflection coefficient before artery closure and the true reflection coefficient; then we can use the true blood pressure to obtain the reflection coefficient. The forward wave and reflection coefficient are used to derive the true blood pressure reflection wave, and finally, by adding the measured PTT parameters, we can reconstruct the true upper arm blood pressure waveform. In this section, two methods are used to reconstruct upper arm blood pressure: Method 1 uses the corrected reflection coefficient of the model in this section to reconstruct upper arm blood pressure, and Method 2 uses the uncorrected reflection coefficient to reconstruct upper arm blood pressure.

The model was divided into three levels, with the first level being the decision level, the second level being the guideline level, and the third level being the protocol level. The investigation carried out in a stepwise manner according to this model hierarchy from top to bottom. That is, the contents of the decision layer were first used as the basis for selecting the guideline layer evaluation score by making judgments by two-two comparisons, and then the contents of each guideline layer were used as the basis for selecting the protocol layer evaluation score, again by making judgments by two-two comparisons, respectively.

Collect clinical and imaging data including age, sex, and other risk factors for cerebrovascular disease and secondary prevention medications, and evaluate the patient’s cranial magnetic resonance images including the specific location of the new infarct in the brainstem, whether it is located in the pars median penetrating artery supply area, whether it is located in multiple groups of brainstem penetrating artery supply areas, the size and volume of the new infarct, the SSS-TOAST classification, the severity of cerebral white matter loss, and the number of asymptomatic old luminal infarcts.

### Analysis of Evaluation Indicators

The data extraction design to produce a uniform data extraction excel sheet required two researchers to independently read the literature, conduct an initial review and evaluation of the title and abstract of each randomized control study, and then read the full text for data extraction. Data extraction included: title of the literature, time of publication, experimental and control group information (total number, number of people, mean age, gender matching, jaded score, outcome measures). The review includes: inclusion and exclusion criteria of the literature, the setting of the control group, if there are disagreements can be discussed and resolved by the two researchers or assisted by other experts or researchers in the subject group, and the two researchers exchange checks after the work is completed. The methodological quality of the study is evaluated by two researchers alone for all included literature, generated from random sequences by applying the Jaded scale; concealment of random assignment scheme, specifically, both the trial implementer and the subjects before grouping The literature quality and methodological quality of the included literature was assessed in four areas: the specific allocation scheme of the subjects could not be known in advance; whether blinding was being used in the study; and whether withdrawal and withdrawal were being used in the study.

The video coding rate distortion algorithm, as a very important part of video coding, has become the subject of many researchers who have come up with many excellent ideas for improving the algorithm, with the ultimate goal of obtaining the best coding mode in which the bit rate R and coding distortion D make the coding cost J minimal. Under the H264 standard is each mode used for the current coding block. The resulting cost calculated for rate-distortion, and then comparative analysis of each mode is performed to pick the least costly class of coding modes, and the selected coding modes are defined as macroblocks to be coded. In H264, the RDO cost function shows that the substitution value is determined by three factors: motion search, reference frame selection, and mode decision, while the standard H264 only uses the traversal calculation method to optimize the rate-distortion, without fully considering the influence of other factors.

**FIGURE 5:**
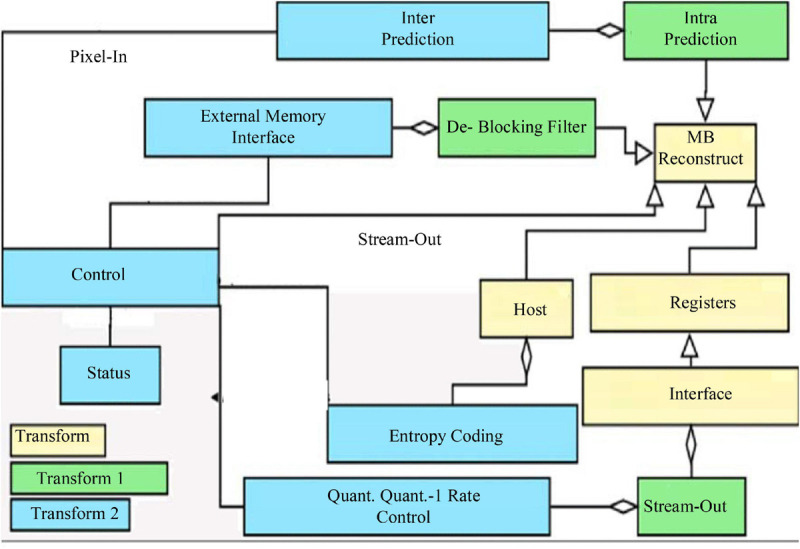
Video image continuity evaluation.

To solve this problem, this paper proposes its improvement ideas on how to confirm the inter-frame macroblock coding mode selection algorithm in the original rate-distortion optimization. There are various ways to divide the inter-frame macroblock, according to the degree of motion we can make different divisions, the large division is suitable for absolute still or relatively still small amplitude motion, a small division is suitable for large changes in position and detail part of the more intense motion. The program finally adopts the 8^∗^8 division of the block to be coded, divides the block into four 8^∗^8 patterns, and analyses the corresponding motion vector direction for the four sub-modules obtained from the 8^∗^8 pattern division. The set of possible coding patterns used in that coding block is then created together to create the coding pattern set and rate-distortion optimization is computed to get the best pattern needed, and tests show that the improved rate-distortion algorithm has improved the coding efficiency, as shown in Figure 5.

As the most accurate search algorithm, FS searches all the search points to get the best match, which is not suitable for transmission in the real-time video due to high computational complexity and can only be used as a standard for comparison with other algorithms. The three-step method is a simplification of the full search method, the fastest case only needs to search 25 search points to get the best match, although the complexity of the calculation is much reduced, and the matching accuracy is also reduced, the three-step method is only suitable for the application of a large range of motion of the frame image, and small-amplitude motion of the frame image, this algorithm is easy to fall into the state of the local optimal solution, which leads to large matching errors. The new three-step method is a supplement to the original three-step method, which makes use of the center bias feature to enhance the matching computation of the central region position, improve the search performance, and has good performance for video sequences with smaller motion. The hexagonal search method and the diamond search method, as the classical block-matching motion estimation algorithms, adopt the same idea and use two different search templates to avoid the defects of local optimization. The algorithm can be regarded as an improvement based on the hexagonal search method and the diamond search method, the hybrid search pattern with multiple templates combined with an early termination strategy can accurately predict the starting search point, which is recognized as the ideal motion estimation algorithm in H264.

## Analysis of Results

### Analysis of Experimental Results

**FIGURE 6:**
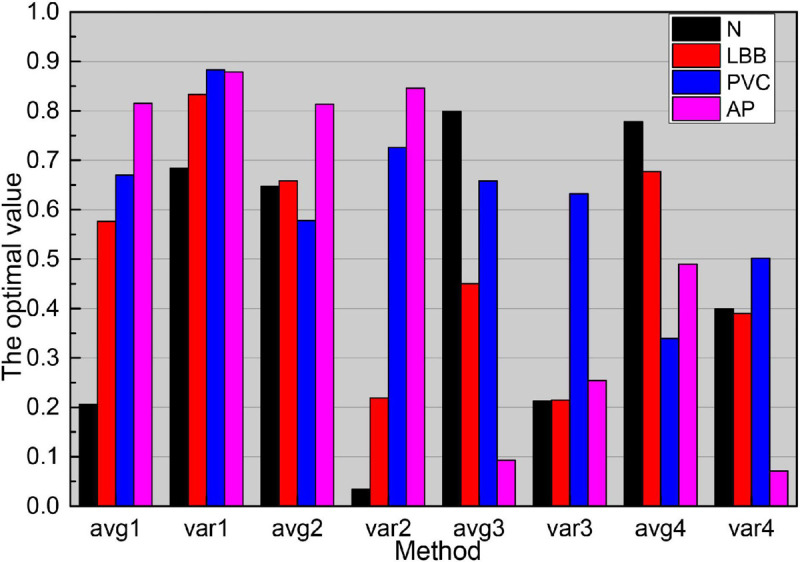
Matching matrix for beat classification.

The matching matrix for the six classes of heartbeat classification is shown in Figure 6, where the rows represent the classification results of the six classes of heartbeats obtained by the algorithm, the columns represent the actual heartbeat classification results, and the data on the diagonal is the number of correctly detected heartbeats in each class of heartbeats. From the data in the table, there are more misidentifications between N beats and AP beats, N beats are easily recognized as AP beats and AP beats are easily recognized as N beats. The main reason is that N and AP beats are similar in morphology, the significant difference lies in the morphological changes of P-wave, which is a wave with a small amplitude, and the changes of P-wave amplitude are relatively large in different individuals, so it is easy to confuse N and AP beats. RBB beats also have some similarities with PVC beats in terms of morphology, which can also lead to misidentification, while P beats have good morphological consistency and are quite different from other types of beats, so P beats have the highest recognition rate.

**FIGURE 7:**
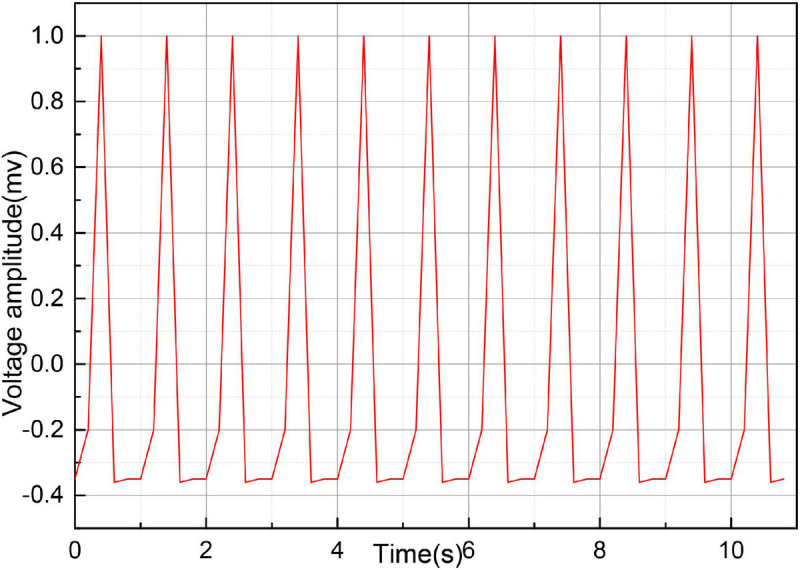
Video image of ECG signal.

Before the onset of SCD, the ECG signal pattern changes accordingly. Figure 7 gives several groups of ECG signal morphology before the occurrence of SCD, the upper subgraph in each figure is the pre-processing signal, and the lower subgraph is the post-processing signal. As can be seen from the figure, although the signal morphology has some changes compared with the normal ECG signal, the morphological changes are not uniform, and the morphological changes are also different in different leads, and even the morphology is not consistent when SCD occurs in different people with the same lead data. In addition to the diversity of SCD evoked and the complexity of the noise contained in the ECG signal, accurate identification of SCD becomes difficult.

**FIGURE 8:**
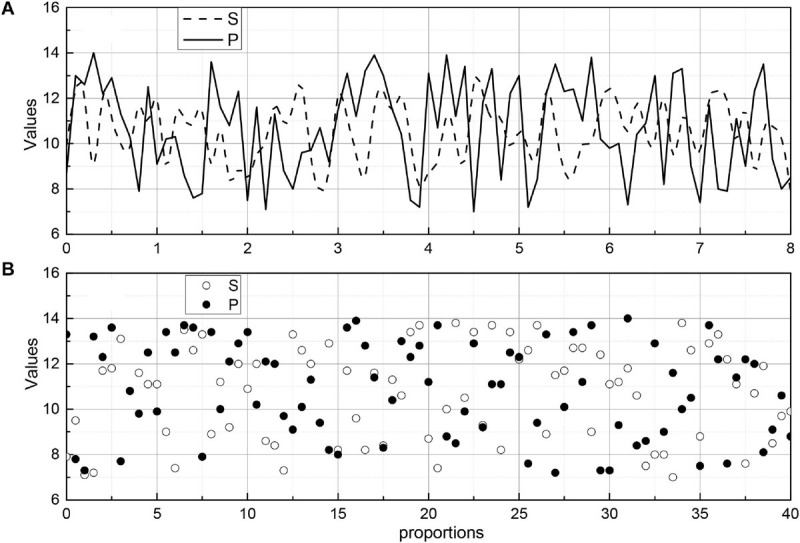
Reserve pool connected spectral radius on the SCD recognition image.

As the main parameters of the ESN network, the reserve pool connection radius, input scaling, and leakage rate have a great influence on the SCD identification results. It can be seen from the data in Figure 8, as the reserve pool connection spectrum radius decreases, its sensitivity, and prediction have increased, and the change is more obvious when it is reduced from 0.1 to 0.01, while when the reserve pool connection spectrum radius is further reduced, S and P no longer improve, instead, there is a significant decrease, so the final setting is 0.001. The input scaling controls the scaling of the input weight matrix. The scaling is too large to significantly reduce the recognition capability of the network, while too small will significantly reduce the recognition sensitivity, and finally 0.1. The leakage rate is the one in folium (4), which controls the rate of dynamic reserve pool update, and with the increasing value of m, the accuracy of SCD recognition is gradually improved until its value is close to 1.

**FIGURE 9:**
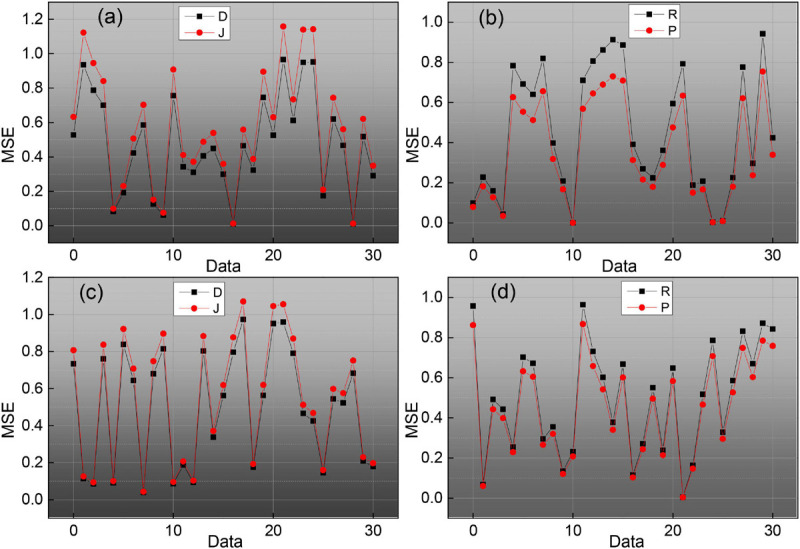
Evaluation index value change curve.

The images were divided into five groups according to the characteristics of the algorithm: normal, border blur, stent, thrombus, and plaque groups. In the normal group, the images had an obvious layered structure with continuous highlighted areas outside the endothelial border; in the fuzzy border group, the images mainly contained those with an obscure layered structure, the similar grayscale value between layers, and extremely fuzzy border or border near the image edge; in the stent group, the images contained stent structure; in the thrombus group, the images contained obvious thrombus or residual blood in the intravascular lumen; and in the plaque group, the images contained various types of the plaque outside the intima-vascular membrane. In each group, 20 sets of images selected for analysis. Figure 9 shows the endothelial extraction results of five groups of typical images, from top to bottom: normal, border blurred, stent, thrombus, and plaque. From left to right, the original images, the gold standard images, and the auto-extraction images of this algorithm are shown respectively. For five groups of typical images, this algorithm can accurately locate the endothelial border position and has a good agreement with the endothelium in the gold standard image.

To evaluate the effect of endomembrane extraction more accurately, we statistically analyzed the average sieve coefficient (D), Jaccard coefficient (J), Harsdorf distance (H), accuracy (P), and recall (R), and the corresponding mean squared deviation for each group of 20 images. At the same time, the change curves of D, J, P, and R for different images in the five sets of images are given, which are shown in Figure 9, where the red line is D, the blue line is J, the green line is P, and the black line is R. We can see the effect of the algorithm on the average sieve coefficient (D), Jaccard coefficient (J), Harsdorf distance (H), accuracy (P), and recall (R), and the corresponding mean squared deviation. We can see that the algorithm in this paper has the best effect on the endosomal extraction of normal images, and several evaluation indexes have good stability for different images. The main reason is that the normal group of images is relatively simple, and the images have high similarity, so it has high precision and stability. For other groups of images, due to the complexity of the image situation, the image itself is quite different, which makes the D, J, P, and R curves change a lot, and the average accuracy is also slightly lower than the normal group.

### Artificial Intelligence Segmentation Dynamic Results Analysis

**FIGURE 10:**
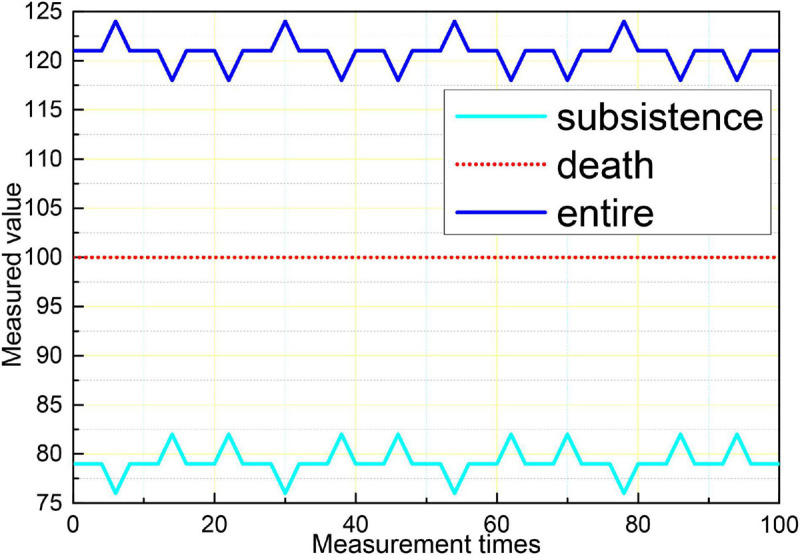
Comparison of the three assessment methods with prognostic logistic regression analysis.

Logistic regression analysis was performed on aEEG classification and prognosis, and the χ^2^-test was performed on the fitness of the regression equation model, *p* = 0.003, indicating that the model fit was good and the regression equation was significant. Logistic regression analysis showed that the accuracy of aEEG classification for survival prognosis was 100%, and the accuracy of the prognosis for death was 42.9%, and the accuracy of the judgment of the comprehensive prognosis was 88.6%. The results of logistic regression analysis showed that the accuracy of the EEG Young classification for survival prognosis was 96.4%, and the accuracy of the regression equation for death prognosis was 88.6%. The accuracy of the prognostic judgment was 57.1 and 88.6% for the combined prognostic judgment. The logistic regression analysis of the GCS score and prognosis showed that the accuracy of the GCS score for survival prognosis was 96.4% and the accuracy of the regression equation for death prognosis was 88.6%. 14.3% and the accuracy of the combined prognostic judgment was 80%, as shown in Figure 10. The results showed that aEEG score and EEG Young score were more accurate than the GCS score for prognostic judgment; and aEEG score combined with EEG Young score could improve the accuracy of prediction.

**FIGURE 11:**
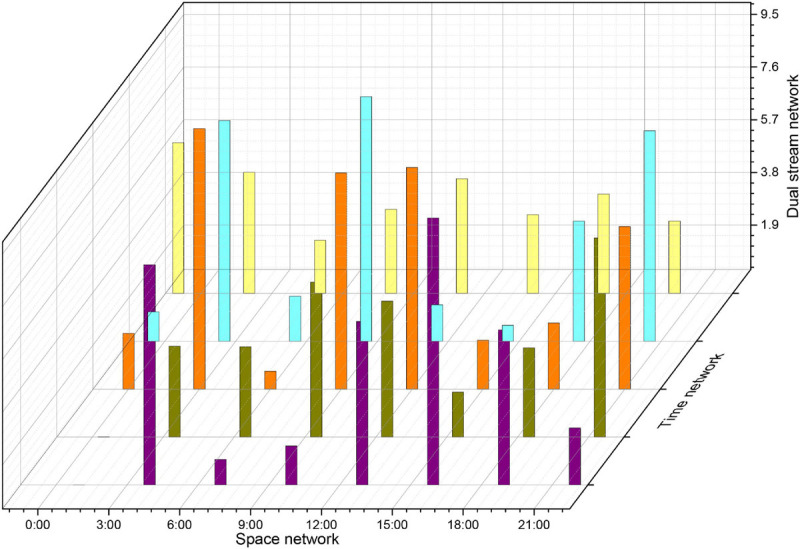
Experimental accuracy on the dataset with different pre-training premises.

To illustrate more clearly the improvement of the effect brought by the method in this paper, this subsection gives a detailed data justification through experiments in terms of the training method, the parameters of the attention mechanism module, the number of fragments and sampling frames, and the effect brought by the fusion method, respectively, and makes a comparison with existing algorithms to analyze the advantages and disadvantages of the model. The training model of a neural network usually has three cases, one is to train from scratch, two is to use ImageNet pre-trained weights and fix the weights before the last layer so that they do not update the parameters, but only the parameters of the network behind, and three is to also use ImageNet pre-trained weights, but the weights are not fixed, but the parameters on the data set to be trained of fine-tuning. The three training methods are all based on the framework, and since the network does not have a pre-trained model, it is not pre-trained to train the spatial and temporal networks separately, and the experimental results are shown in Figure 11. As can be seen in Figure 11, the third training method has the highest accuracy, training from zero has the worst effect, and fixing the previous parameters has the second-worst effect because training from zero is more difficult in the first place, it will take longer to converge when the gradient is updated, and it can converge to a satisfactory effect when the amount of data is sufficient, but the sample size of the dataset is small, only close to 10,000 training samples, so pre-training weights are needed, because ImageNet is a very large image dataset, on top of this training network weights for small datasets will speed up convergence, and improve accuracy, fixed pre-training weights, only update the network behind, but also compared to training from scratch has good results. So there are three ways to classify different scenarios and strengths, the first way to use the data set is relatively large, and no pre-training framework, this method of training convergence is slower, requiring a long time to see iterations to achieve better results for small data sets, this way the results are poor; the second way for small data sets, because the network parameters are large, small data sets cannot be on the network parameters are trained completely, in which case fixing the weights trained by ImageNet and updating only the last classification layer tends to achieve better results; the third way applies to cases such as data sets, which can be fine-tuned on the data set to be trained to achieve better accuracy and to speed up the convergence of the network. Given the above considerations, the third pre-training approach is chosen for all subsequent experiments.

**FIGURE 12:**
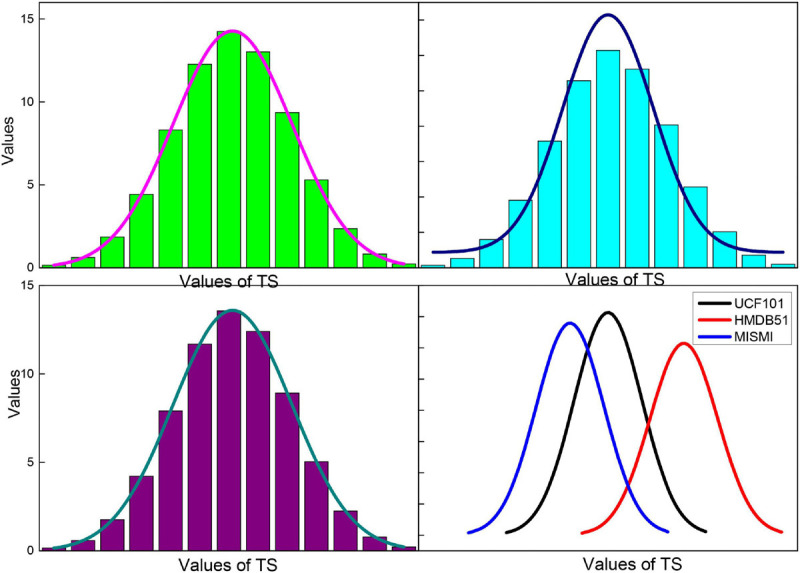
Image recognition accuracy of the method in this paper for different TS values.

The choice of the parameter time step of the attention mechanism module and its corresponding LSTM in the approach presented in this paper has a significant impact on the performance of the attention mechanism. The experiments in this section evaluate the accuracy of the action recognition obtained by the method at different TS values from 1 to 20, and the results on the databases are shown in Figure 12. When TS = 1, the distribution of attentional weights in 30 frames can be learned in only one iteration while fitting the distribution of importance in only 30 frames is not sufficient. As the TS value increases, the recognition accuracy gradually improves. When TS = 10, the proposed method achieves the highest recognition accuracy of 94.4 and 71.5% on the databases, respectively. As the value of TS continues to increase, the accuracy of action recognition decreases rapidly after remaining stable for some time. This is because as the TS value continues to increase, the size of the trainable parameters of the expanded LSTM increases rapidly, which leads to overfitting. Therefore, the time step TS of the attentional mechanism is determined to be 10 in this paper’s approach.

**FIGURE 13:**
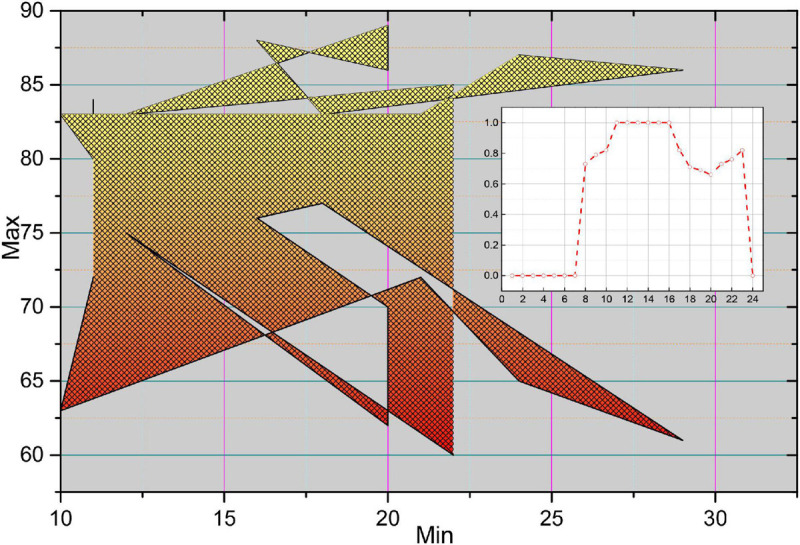
Unfolded image and extract A-line.

To reduce the interference of catheter rings, residual blood in the vessel, or thrombus during A-line modeling, we use the endovascular boundary obtained by the algorithm in the previous chapter to expand the OCT image along the endovascular boundary. The specific implementation steps are to scan each column of the OCT image, detect the endothelial border points, and select the next point as the starting point of the column, and then take the points downward, a total of 100 pixels so that the OCT image is expanded along the endothelial border, as shown in Figure 13.

**FIGURE 14:**
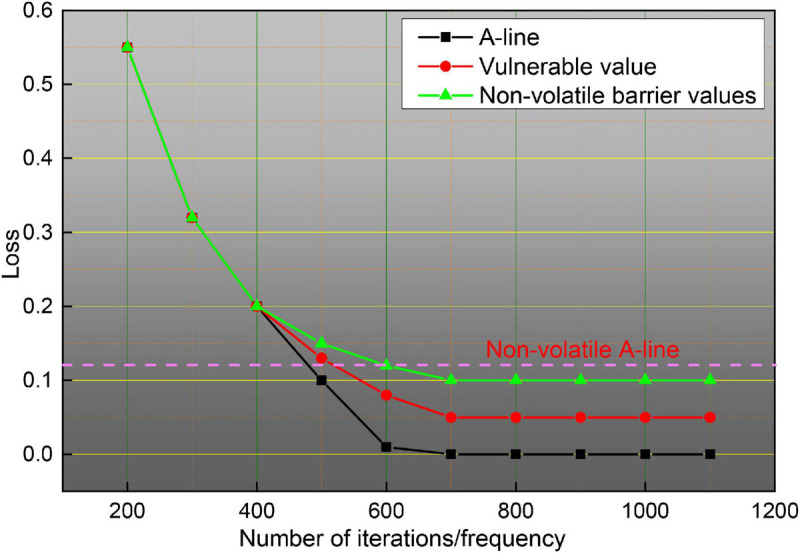
Perishable and non-perishable A-line.

In the unfolded OCT image, each column element constitutes an A-line, which has different morphological characteristics of F due to the light absorption property of the plaque itself and the position relation with the intima. Figure 14 shows four typical A-line morphologies, in which the red line is F, the green line is F-C, the blue line is F-L, and the pink line is GS. Due to the clear border of calcified plaques, the A-line of F-C plaques will have a more drastic change in slope; due to the blurred border of F-L plaques, the A-line of F-L plaques will usually be relatively flat, and there will be a staggered distribution of high and low values; while the A-line of GS plaques is close to the zero line, and there may be small fluctuations at the starting position.

In this paper, we focus on intelligent analysis methods for high-risk cardiovascular diseases, making full use of the portable and long acquisition time features of dynamic ECG to study the intelligent prediction algorithm for arrhythmias and SCD at the level of ECG signal. A sparse auto-coding deep neural network with a four layer stack structure was constructed to automatically extract the depth features of arrhythmia beats, the automatic recognition of six classes of beats was achieved by SoftMax classifier, the training process of the network was optimized by a parsimonious Newtonian optimization algorithm so that the extracted depth features could more accurately describe the input signal, and the key parameters of the network were discussed and analyzed. By taking full advantage of the recognition ability of the echo state network on the time domain signal, the ESN network with a multilayer tandem structure was designed to achieve accurate recognition of the sudden cardiac death signal. The accurate predictions were 93.04, 95.36, 94.20, 94.20, and 94.78%, respectively, when tested using the signal 5 min before the onset of sudden death.

## Conclusion

In this paper, the continuity of segmented dynamic video images in the detection of severe cardiovascular and cerebrovascular diseases is investigated and analyzed by artificial intelligence, taking full advantage of the high precision and high resolution of medical images. Based on the characteristics of different OCT images containing different tissue information with different gray level distribution, and automatic gray level label value selection method based on image gray level distribution characteristics developed to ensure the stability of the algorithm in the extraction of endosomes from different OCT images. The effect of the algorithm on the endomembrane extraction under different disturbances is also verified. Fully utilizing the self-learning ability of stack sparse automatic coding network for label-free data, the deep modeling analysis of A-line in OCT images was performed to achieve accurate identification of fibro genic, fibro genic-calcified, and fibro genic-lipid A-line, and the automatic extraction of plaque regions based on the automatic plaque region generation algorithm. On this basis, the automatic identification of thin fibrous cap-like vulnerable plaques was achieved by analyzing the fibrous cap thickness. In this paper, the algorithm achieved simultaneous automatic recognition of plaque and vulnerable plaque by modeling and analysis of A-line in OCT images, with recognition accuracy, recall rate, and area overlap of 84.29, 84.29, and 87.33%, respectively.

## Data Availability Statement

Publicly available datasets were analyzed in this study. This data can be found here: http://epileptologie-bonn.de/cms/upload/workgroup/lehnertz/eegdata.html.

## Author Contributions

All authors listed have made a substantial, direct and intellectual contribution to the work, and approved it for publication.

## Conflict of Interest

The authors declare that the research was conducted in the absence of any commercial or financial relationships that could be construed as a potential conflict of interest.
